# Prehospital Predictors of Survival in Patients with Out-of-Hospital Cardiac Arrest

**DOI:** 10.3390/medicina59101717

**Published:** 2023-09-26

**Authors:** Matej Strnad, Vesna Borovnik Lesjak, Pia Jerot, Maruša Esih

**Affiliations:** 1 Prehospital Unit, Center for Emergency Medicine, Community Healthcare Center, Cesta Proletarskih Brigad 21, 2000 Maribor, Slovenia; vesnick@gmail.com; 2 Emergency Department, University Medical Center Maribor, Ljubljanska ul. 5, 2000 Maribor, Slovenia; esih.marusa@gmail.com; 3 Department of Emergency Medicine, Medical Faculty, University of Maribor, Taborska 8, 2000 Maribor, Slovenia; 4 Community Healthcare Center, Mariborska Cesta 37, 2360 Radlje ob Dravi, Slovenia; piajerot@gmail.com

**Keywords:** automated external defibrillator, cardiopulmonary resuscitation, out-of-hospital cardiac arrest, response time, sudden cardiac death, survival rate, ventricular fibrillation

## Abstract

*Background and Objectives*: Despite advances in the treatment of heart diseases, the outcome of patients experiencing sudden cardiac arrest remains poor. The aim of our study was to determine the prehospital variables as predictors of survival outcomes in out-of-hospital cardiac arrest (OHCA) victims. *Materials and Methods*: This was a retrospective observational cohort study of OHCA cases. EMS protocols created in accordance with the Utstein style reporting for OHCA, first responder intervention reports, medical dispatch center dispatch protocols and hospital medical reports were all reviewed. Multivariate logistic regression was performed with the following variables: age, gender, witnessed status, location, bystander CPR, first rhythm, and etiology. *Results*: A total of 381 interventions with resuscitation attempts were analyzed. In more than half (55%) of them, bystander CPR was performed. Thirty percent of all patients achieved return of spontaneous circulation (ROSC), 22% of those achieved 30-day survival (7% of all OHCA victims), and 73% of those survived with Cerebral Performance Score 1 or 2. The logistic regression model of adjustment confirms that shockable initial rhythm was a predictor of ROSC [OR: 4.5 (95% CI: 2.5–8.1)] and 30-day survival [OR: 9.3 (95% CI: 2.9–29.2)]. Age was also associated (≤67 years) [OR: 3.9 (95% CI: 1.3–11.9)] with better survival. *Conclusions*: Elderly patients have a lower survival rate. The occurrence of bystander CPR in cardiac arrest remains alarmingly low. Shockable initial rhythm is associated with a better survival rate and neurological outcome compared with non-shockable rhythm.

## 1. Introduction

In Europe, out-of-hospital cardiac arrest (OHCA) annually affects around 275,000 people and is one of the leading causes of death [1,2].

Despite advances in the treatment of heart diseases, the outcome of patients experiencing sudden cardiac arrest remains poor [3,4]. Even though bystander cardiopulmonary resuscitation (CPR) and time to defibrillation have improved, we are dealing with increasing age of patients with OHCA and a decreasing proportion of them presenting with ventricular fibrillation [3]. The aggregate survival rate of OHCA patients recorded across various populations is between 6.7% and 8.4% [5].

Studies have evaluated the strength of associations between OHCA and key factors that have an impact on survival rate [3,5] and found that cardiac arrest witnessed by a bystander or by an emergency medical service (EMS), provision of bystander CPR [3], shockable initial cardiac rhythm [3,5], and return of spontaneous circulation (ROSC) in the field are associated with better survival [2,3,5].

CPR is an emergency lifesaving procedure and can double or triple the chance of survival from OHCA [6]. The survival rate is also associated with the etiology of sudden cardiac arrest and the first monitored rhythm. When the initial rhythm is asystole, the likelihood of successful resuscitation is low [3]. Forty percent of patients with OHCA are found with initial shockable rhythms, yet only 22% of them achieve ROSC. This group may be a priority population for future efforts to improve ROSC and survival of OHCA patients [5].

The aim of the present observational study was to determine the prehospital variables that serve as predictors of survival outcomes in OHCA victims.

## 2. Methods

The regional ethics committee of University Medical Center Maribor and Community Healthcare Center Maribor approved the study and waived the requirement to obtain any informed consent (No. UKC-MB-KME-24/20, 02/010/03-003/01/20, respectively).

### 2.1. Setting

This study was conducted in the city of Maribor, Slovenia and adjacent rural areas encompassing a population of app. 200.000 inhabitants spread over an area of app. 780 km^2^.

### 2.2. Emergency Medical Services (EMS) and Study Design

In Slovenia, EMSs are provided according to the Rules on Emergency Medical Service by the Community healthcare centers with EMS stations situated in urban areas. The catchment area of a single EMS expands over several surrounding municipalities. The Maribor EMS system includes two advanced life support (ALS) teams and four basic life support (BLS) teams, and during the daytime from May to November, a BLS rescuer on motorcycle. An ALS team consists of two rescuers (registered nurse and medical technician) and an emergency physician on board. A BLS team includes a registered nurse and a medical technician. All rescuers in the EMS are required to obtain a National Vocational Qualification Certificate in accordance with the Rules on Emergency Medical Services published in the Official Gazette of the Republic of Slovenia. When a cardiac arrest is suspected, an ambulance with the ALS team on board is dispatched to the scene. If the two ALS teams are responding to other emergencies, a BLS team is dispatched first. The motorcycle rescuer is also dispatched, arriving typically first to the scene.

In the rural areas of EMS Maribor (response time > 10 min), a dual dispatch system of the first responder system had been organized gradually since May 2014 and became fully operational in the beginning of 2015. Volunteer firefighters who want to become first responders are required to complete a 10 h training course to learn the following basic life support (BLS) competences: adult and pediatric CPR using an automated external defibrillator (AED), foreign body airway obstruction, and control of the major isolated traumatic external bleeding. The first responder license requires passing both a written and a practical exam after the training. Annual license renewal is required for all first responders (a more in-depth explanation of the first responder system and dual dispatch system can be found elsewhere) [7].

This was a retrospective observational cohort study of the prospectively gathered data. For the duration of the study, EMS protocols created in accordance with the Utstein style reporting for OHCA, first responder intervention reports, medical dispatch center dispatch protocols and hospital medical reports were all reviewed.

### 2.3. Study Participants

All EMS-resuscitated OHCA victims were eligible (regardless of witnessed status, cardiac rhythm or prior morbidity). Patients aged ≤18 years, patients with overt clinical signs of irreversible death, those with existing Do Not Attempt Resuscitation (DNAR) orders and patients where no CPR was initiated by the EMS were excluded.

### 2.4. Endpoints

The primary endpoints were: (1) number of OHCA patients with ROSC, (2) 30-day survival and (3) number of patients with good neurological outcome (Cerebral Performance Score (CPC) 1 or 2). Secondary endpoints were: (1) response times and (2) proportion of patients presenting with VF/VT as initial rhythm.

### 2.5. Statistical Analyses

Frequency distributions (means, standard deviation, and percentages) are used to present the characteristics of survivors after OHCA. Comparisons between different groups were analyzed with t-test for age and Mann-Whitney U test for categorical data. Multivariate logistic regression was used to control for confounding factors affecting the outcomes (ROSC and 30-day survival). In a multivariable model, the following variables were added: age, gender, witnessed status, location, bystander CPR, first rhythm, etiology. Associations are presented as odds ratios (ORs) with 95% confidence intervals (CIs). Results were regarded significant if a two-tailed test yielded a *p*-value equal to or less than 0.05. For statistical analysis, SPSS software (version 25, SPSS Inc., Chicago, IL, USA) was used.

## 3. Results

A total of 381 patients who were resuscitated were included in this study. Of those, 30% of patients achieved ROSC, 22% of those survived until 30 days after cardiac arrest (7% of all OHCA victims), and 73% of those survived with CPC 1 or 2. Baseline characteristics of patients who achieved ROSC and their effect on primary outcomes are introduced in Table 1.

Nearly a quarter (23%) of patients included in our study experienced cardiac arrest outside of home. More than half (55%) of them received bystander CPR and 21% of them had a good 30-day survival rate.

The data on comorbidities were obtained retroactively through the hospital’s database for all 351 patients. For 31 of them, the data of comorbidities were not available. Out of 351 patients, 73% of them had one or more comorbidity. The majority of them (46.3%) had known arterial hypertension. The second most common comorbidity was diabetes (19.9%). Up to 20% of patients included in the study who experienced cardiac arrest had a known chronic heart failure (NYHA 3 and 4) or coronary heart disease. Pulmonary disease was diagnosed in 9.4% of patients and 8.9% of patients had a known peripheral arterial disease. 7.3% of all patients had a known malignancy at the time of cardiac arrest. Drug abuse or psychiatric disease were less common with 7.1%. Smoking as a comorbidity and known risk factor for acute coronary syndrome and other vascular diseases was present in 3.9% of all enrolled patients. One or more comorbidities were present in 61.1% of patients younger than 68 years and in 79.9% of patients 68 years old or older (*p* = 0.007).

Based on the above findings and previous reports, we included potential influencing factors into a multivariate logistic regression analysis on ROSC and 30-day survival. Cardiac etiology had no statistically significant impact on ROSC (ROSC was achieved in 29.7% in the cardiac etiology group and in 33.3% in the group with noncardiac or other etiology). However, all patients that survived until 30 days had an arrest of cardiac etiology. Results for other factors are given in Table 2.

With special attention to the presence of bystanders and their CPR performance, we compared patient survival between those who received bystander CPR and those who did not (Table 3).

## 4. Discussion

Findings from our study suggest that a better survival rate after OHCA is associated with younger age and shockable first rhythm.

### 4.1. Bystander CPR

Wissenberg et al. examined the temporal trends in bystander CPR rates and the survival outcomes between the years 2001 and 2010 in Denmark. During the mentioned period, various national initiatives were launched to improve bystander resuscitation rates and advanced care. There was an increase in bystander CPR rates from 22.1% (2001) to 44.9% (2010) and the increase in bystander CPR rates was significantly associated with survival on arrival at hospital, 30-day survival and 1-year survival in OHCA patients [8]. Survival after OHCA is greater among those who received bystander CPR immediately after the cardiac event compared to those who received delayed CPR from EMS. Bystander CPR while waiting for an ambulance was associated with a 2.3-fold increase in 30-day survival at 5 min and a 3.0-fold increase at 10 min [9,10]. A study also showed higher chances of survival for patients who received bystander CPR and first responder or bystander defibrillation compared with patients who received bystander CPR and defibrillation later by the medical personnel [10,11]. Furthermore, earlier CPR and prompt defibrillation are associated with less damage to the central nervous system and other organs, including the heart [3,9]. These findings are supported by our data that show that bystander CPR corresponds with a tendency toward better neurologic outcome in OHCA patients.

Even though bystander CPR has a great benefit for survival of OHCA patients, it is not always performed. The main reasons are concern of doing something wrong, lack of knowledge and concerns about possible transmission of disease while performing rescue breathing [3,12]. Our study showed that out of 352 witnessed cardiac arrests, regardless of location, only 146 people received CPR and 136 did not, which is very concerning. The same poor implementation of CPR knowledge among lay people in Slovenia was confirmed with the study by Rajapakse and colleagues in 2006 regarding knowledge about BLS. Less than half of participants knew that CPR consisted of rescue breathing and chest compressions. Only 1.2% knew the correct rate of chest compressions, 2.2% knew the correct compressions-to-ventilations ratio in adult CPR, and only three out of 500 subjects (0.6%) knew both. Study showed that knowledge about cardiac arrest and CPR is not sufficient, but it is markedly better in those who received previous CPR training [13]. In contrast, in Norway, where first aid has been taught in schools since 1961 and is a part of the current school curriculum for primary and lower secondary schools [14], theoretical knowledge of handling an adult who appears to be unresponsive was high and 90% of participants knew the national medical emergency telephone number (113). Additionally, the majority (83%) of the participants were willing to perform BLS in case of cardiac arrest [15]. CPR training in schools enables education of a wide population and presents a vital part of knowledge dissemination fostering positive attitude toward CPR among nonprofessionals [8,16].

Other concerns regarding performance of CPR by bystanders are fear of causing injury and inappropriate contact due to sexualization of women’s bodies [17]. According to reports from different studies, women are less often resuscitated by bystanders than men [17,18], which was also confirmed by our results but with a much smaller difference between the sexes (48% women received bystander CPR vs. 56% men) compared to other studies [17]. Based on the mentioned results, it could be assumed that sexualization of the female body is less pronounced in Slovenia compared to other countries where the difference in bystander CPR regarding sex is much higher [17,18].

Studies have shown that patients collapsing outside of the home are more frequently men and when cardiac arrest outside of the home is more often witnessed, patients are more likely to receive bystander CPR, mean time to CPR is shorter, and the first-monitored rhythm is more frequently ventricular fibrillation. Based on these facts, survival from cardiac arrest is higher when the collapse occurs outside of the home [19]. Nearly a quarter (23%) of patients included in our study experienced cardiac arrest outside of the home. More than a half (55%) of them received bystander CPR and 21% of them survived until 30 days. On the other hand, 53% of patients who collapsed at home were resuscitated by a bystander but only 10% of them survived until 30 days.

### 4.2. First Responders and AED Usage

Because of the importance of a swift prehospital response when treating OHCA patients and to increase the likelihood of immediate CPR and rapid defibrillation with an AED, systems that dispatch First Responders (FRs) have been developed all over Europe. Over 50% of European countries have a dispatched FR system to respond to a suspected OHCA in place [20].

First responders mostly consist of volunteer firefighters, police officers and citizen-responders [20,21].

First responders (local volunteer firefighters) have been organized in Slovenia in the rural areas where the EMS response time exceeds 10 min. First responders’ response times are significantly shorter compared to EMS response times, thereby shortening the time to initiation of CPR and prompt AED use [21,22,23,24]. A study from Rajan et al. showed that immediate defibrillation is useful if provided within 4 min of cardiac arrest [10]. Another study by Karlsson et al. demonstrated that early defibrillation with an AED can improve survival from an OHCA to >50% and reduce the risk of anoxic brain injury [25]. Of great importance is the correct use of an AED among first responders and also among lay people. AED skills by lay people seem to be associated with younger age and prior attendance to CPR courses. However, in a study by Stropnik and Klemenc-Ketiš, only 2% of participants correctly performed all five steps of defibrillation with an AED [26]. The poor knowledge of AED use was confirmed in our study. Only one bystander correctly used an AED. The low incidence could be explained by poor accessibility of AEDs, which greatly affects its use.

According to the study by Karlsson et al. mentioned above, having access to a nearby and available AED tripled (13.8% vs. 4.8%) the likelihood of bystander defibrillation and nearly doubled (28.8% vs. 16.4%) 30-day survival [25]. AED accessibility was highly dependent on AED location; it was concluded that the most frequent locations of AED do not have 24/7 accessibility (shops, pharmacies, schools etc.) [10,25].

Slovenia does not have an accurate official AED network, which could be available to bystanders as first responders. The other major problem is that the dispatch center has no insight into the layout or the location of AEDs, which are most often placed in pharmacies, shopping centers, fire stations, etc., which are not open 24/7 and so AEDs are not available for use by bystanders, especially when cardiac arrest takes place at home. If the Dispatch center had its own database of AED locations, it would be able to direct the bystanders calling towards the nearest AED, thus reducing the time to the first defibrillation. The issue with AEDs in Slovenia is also their placement, which is decided solely by the AED owner, resulting in a random distribution of AEDs, which could be improved.

The means to promote the use of AEDs by lay persons in OHCA include increasing public awareness about the AED network and informing them about the nearest location of accessible AED [27].

### 4.3. Shockable vs. Non-Shockable Cardiac Rhythm

During the past two decades the incidence of OHCA patients presenting with shockable rhythms has decreased [28].

There is a known relation between non-shockable rhythms and poor success rates of resuscitation [28,29]. Thirty-day survival is significantly higher in patients with converted shockable rhythms than patients with sustained non-shockable rhythms [30].

A shockable initial rhythm is associated with a better chance of a good neurological outcome and 30-day survival in refractory OHCA [31]. Patients who present with shockable rhythms represent a majority of all cardiac arrest survivors probably due to a high prevalence of reversible cardiac causes of arrest. The vast majority of cardiac arrests with ventricular fibrillation as the first rhythm are related to an underlying cardiac disorder. Cardiac causes are less frequent in patients with asystole or pulseless electrical activity (PEA) [30,31].

In accordance with previously mentioned studies, data from our study endorsed the association between shockable initiable rhythm and ROSC and 30-day survival. Half of all patients with cardiac arrest who gained ROSC presented with shockable initial rhythm. In addition, a higher 30-day survival rate of those with a shockable initial rhythm was proved.

### 4.4. Age

Wissenberg and colleagues showed that long-term survival rates after OHCA were less pronounced with increasing age, especially in patients older than 80 years. The explanation can be a lower physiological reserve and presence of comorbidities in patients of older age [32]. In accordance with this and other studies our data confirmed higher incidence of comorbidities and lower survival rates of patients who are aged 68 years or more.

## 5. Conclusions

Elderly patients have a lower survival rate. The majority of patients experienced cardiac arrest at home but less than half of them received bystander CPR. The incidence of bystander CPR in cardiac arrest remains alarmingly low and further efforts to improve BLS knowledge and AED use are most needed. Shockable initial rhythm is associated with a better survival rate and neurological outcome compared with non-shockable rhythm.

### Study Limitations

Our study has several limitations. Firstly, because it is a retrospective analysis of prospectively collected data, some bias could occur. Results gathered from registry data could be limited by missing or incorrectly reported variables. Secondly, the study design makes it difficult to draw accurate conclusions about causality. Thirdly, the population included in our study is small, so the data are not as representable as those in major studies where a number of OHCA patients is much higher. Fourthly, modifications to pre- and in-hospital treatment guidelines that occurred through the study period may have had an impact on the results.

## Figures and Tables

**Table 1:** Characteristics of survivors after OHCA.

	ROSC (*n* = 116)	*p*	30-Day Survival (*n* = 26)	*p*	CPC 1 or 2 (*n* = 19)	*p*
Mean age in years (SD)	61.5 (16.3)	0.002	53 (16.8)	<0.001	54.3 (15.8)	0.242
Male gender, *n* (%)	79 (68.1)	0.089	19 (73.1)	0.955	14 (73.7)	0.549
Location of collapse *At home, n (%)* *At work, n (%)* *Place of sports/recreation, n (%)* *Street/highway, n (%)* *Public building, n (%)* *Residential care/nursing home, n (%)* *Educational institution, n (%)* *Other, n (%)*	78 (69) 5 (4.4) 1 (0.9) 11 (9.7) 6 (5.3) 6 (5.3) / 6 (5.3)	0.023	15 (60) 1 (4) 1 (4) 2 (8) 2 (8) 2 (8) / 2 (8)	0.030	10 (55.6) 1 (5.6) 1 (5.6) 2 (11.1) 2 (11.1) 1 (5.6) / 1 (5.6)	0.322
Witnessed collapse, *n* (%)	102 (88.7)	0.010	23 (92)	0.105	17 (89.5)	0.497
Bystander CPR performed, *n* (%)	60 (56.1)	0.576	14 (58.3)	0.696	13 (68.4)	0.329
1st rhythm shockable, *n* (%)	49 (43)	<0.001	18 (72)	<0.001	13 (72.2)	0.756
Cause *Cardiac/medical, n (%)* *Trauma, n (%)* *Overdose, n (%)* *Submersion, n (%)* *Electrocution, n (%)* *Asphyxia, n (%)* *Unknown??, n (%)*	89 (79.5) 6 (5.4) 1 (0.9) 2 (1.8) 4 (3.6) 4 (3.6) 6 (5.4)	0.550	26 (100) / / / / / /	0.013	19 (100) / / / / / /	/
Comorbidities	84 (77.8)	0.191	15 (68.2)	0.678	10 (66.7)	0.573
Time from arrest to arrival of EMS, min (SD)	8.2 (11.8)	0.405	8.8 (5.6)	0.964	8.6 (6)	0.381
Time from arrest to arrival of FR, min (SD)	7.7 (4.7)	0.872	4.7 (4.2)	0.232	4 (5.7)	/
Time to first shock, min (SD)	11.7 (7)	0.176	11.5 (6.9)	0.430	11.6 (7)	0.335

Note: SD, standard deviation; CPR, cardiopulmonary resuscitation; CPC, Cerebral Performance Category; ROSC, return of spontaneous circulation; FR, first responders; EMS, Emergency medical services.

**Table 2:** Multivariate logistic regression analysis on factors associated with ROSC (*n* = 116) and 30-day survival (*n* = 26).

		ROSC (%)	Adjusted OR	95% CI	30-Day Survival (%)	Adjusted OR	95% CI
Bystander CPR	Yes	31.6	1.127	0.670–1.896	0.1	1.470	0.515–4.195
	No	28.8			0.1		
Age	≤67 years	33.3	1.348	0.794–2.288	0.1	3.907 *	1.279–11.931
	≥68 years	30			0.3		
Gender	Female	62.6	2.119 *	1.193–3.763	0.1	1.936	0.618–6.061
	Male	28.2			0.1		
Witnessed status	Yes	33.3	2.027	0.961–4.275	0.1	4.172	0.503–34.575
	No	17.8			0		
Location	At home	37	0.499 *	0.274–0.910	0.1	0.166 *	0.054–0.508
	Other	2.5			12.9		
First rhythm	Shockable	50	4.520 *	2.535–8.062	21.9	9.337 *	2.983–29.221
	Non-shockable	23.3			0		

Notes: *, *p* < 0.05; OR, odds ratio; CPR, cardiopulmonary resuscitation; ROSC, return of spontaneous circulation; CI, confidence interval.

**Table 3:** Survival of patients after ROSC in regards to bystander CPR performance.

	N of All Documented Cases (Bystander CPR/ No Bystander CPR)	Bystander CPR Group	No Bystander CPR Group	*p*
ROSC	190/163	60 (31.6%)	44 (28.8%)	0.576
*Location AT HOME*	187/161	139 (74.3%)	125 (77.6%)	0.473
*Cardiac etiology*	185/156	150 (81.1%)	129 (82.7%)	0.701
*Witnessed*	190/162	146 (76.8%)	136 (84%)	0.096
*VF/VT*	189/160	54 (28.6%)	41 (25.6%)	0.538
Survival to HA	186/162	51 (27.4%)	46 (28.4%)	0.840
30-day survival	175/146	14 (8%)	10 (6.8%)	0.697
Survival CPC 1/2	13/9	12 (92.3%)	7 (77.8%)	0.340

Note: CPR, cardiopulmonary resuscitation; CPC, Cerebral Performance Category; ROSC, return of spontaneous circulation; VF/VT, ventricular fibrillation/ventricular tachycardia; HA, hospital admission.

## Data Availability

The data used to support the findings of this study are available from the corresponding author upon request.
